# Clinical features and risk factors for primary Sjögren’s syndrome combined with interstitial lung disease: a retrospective study

**DOI:** 10.3389/abp.2024.12461

**Published:** 2024-04-02

**Authors:** Zhixia Yang, Hao Zhao, Lei Shan, Dan Wang

**Affiliations:** ^1^ Department of Rheumatology, Yueyang Hospital of Integrated Traditional Chinese and Western Medicine, Shanghai University of Traditional Chinese Medicine, Shanghai, China; ^2^ Department of Emergency, Shanghai Traditional Chinese Medicine—Integrated Hospital, Shanghai University of Traditional Chinese Medicine, Shanghai, China

**Keywords:** primary Sjögren’s syndrome, interstitial lung disease, clinical characteristics, risk factors, diagnosis

## Abstract

**Objective:**

To analyze the clinical characteristics of primary Sjögren’s syndrome (pSS) combined with interstitial lung disease (ILD), so as to provide a theoretical basis for the early diagnosis, treatment and prevention of PSS-ILD.

**Methods:**

From October 2017 to January 2022, patients with pSS who were admitted to the Department of Rheumatology at Yueyang Hospital of Integrated Traditional Chinese and Western Medicine, Shanghai University of Traditional Chinese Medicine were included in this retrospective study. Patients were divided into the pSS-ILD (102 cases) and pSS-non-ILD groups (154 cases) based on the presence or absence of ILD on high-resolution computed tomography (HRCT). Demographics information, clinical symptoms, laboratory indicators and HRCT features were compared, and the logistic regression analysis was utilized to identify the risk factors.

**Results:**

A total of 256 patients were included. Patients with pSS-ILD were more often female, and their age and disease duration were significantly higher than those in the pSS-non-ILD group (*p* < 0.05). The HRCT imaging classification included ground glass-like shadow (78.4%) and patchy solid shadow (17.6%), and Non-specific interstitial pneumonitis (NSIP) (72.5%) was the predominant typology. Regarding the laboratory indexes, the positive rates of erythrocyte sedimentation rate, C-reactive protein, white blood cell count, neutrophil/lymphocyte ratio, triglycerides, total cholesterol, and anti-SS-A52 antibodies were significantly higher in the pSS-ILD patients than in the pSS-non-ILD group, while the positive rates of anti-synaptic antibodies were lower than in the pSS-non-ILD group, and the differences between two groups were statistically significant (*p* < 0.05). Logistic regression showed that age >60 years, longer duration of disease, higher triglycerides, and cholesterol were risk factors for pSS-ILD patients.

**Conclusion:**

The clinical features of pSS-ILD patients were xerophthalmia, cough and shortness of breath, and HRCT can help to diagnose the disease at an early stage. Age over 60 years, chronic course of disease, and elevated lipid levels are risk factors for ILD in pSS patients, and the relationship between autoimmune antibody levels and the occurrence of ILD needs to be further confirmed in follow-up studies with large sample sizes. These findings have the potential to provide useful information for early diagnosis, treatment, and prevention of the development of pSS-ILD.

## Introduction

Primary Sjogren’s syndrome (pSS) is a common chronic autoimmune disease with a prevalence of 0.33%–0.77%, which is characterized by exocrine lymphocyte infiltration, mainly involving salivary glands and lacrimal glands (Zhang et al., 1993; Mariette and Criswell, 2018). Due to the presence of multiple autoantibodies in the patient’s serum, pSS is a complex heterogeneous autoimmune disease with a diverse clinical face, with almost three-quarters of patients developing extraducular lesions, including the lungs, kidneys, liver, and nervous system (Martin-Nares and Hernandez-Molina, 2019; Yayla et al., 2020; Manfre et al., 2022). Pulmonary lesions are one of the most common extraglandular manifestations of pSS, with an incidence of 9%–75% (Palm et al., 2013; Reina et al., 2016; Manfredi et al., 2017; Roca et al., 2017; Lee et al., 2021), of which the incidence of Interstitial Lung Disease (ILD) can reach 30.1% (Zhao et al., 2020). Moreover, pulmonary involvement is closely related to the prognosis of pSS patients. Compared to pSS patients without lung involvement, patients with lung involvement have a significantly lower quality of life (Dogru et al., 2017). It is reported that patients with pSS-ILD have a mortality rate of up to 39% and a 4-fold increase in mortality after 10 years (Parambil et al., 2006; Palm et al., 2013). Therefore, early screening of patients with high risk for the development of ILD is of great significance for prompt intervention in order to improve prognosis.

Prior studies have found that male gender, older age, and elevated levels of C-reactive protein (CRP) are risk factors associated with the development of interstitial lung disease in patients with primary Sjögren’s syndrome (He et al., 2020). However, there is still limited reporting on the comprehensive assessment of ILD in pSS patients, which combines the patients’ immune antibody levels and high-resolution computed tomography (HRCT) manifestations. Thus, this study aimed to analyzed the clinical characteristics of pSS-ILD and identified the risk factors related to the development of interstitial lung lesions in pSS patients.

## Materials and methods

### Study design and participants

This retrospective study started with identification of pSS patients by searching the electronic medical records. We enrolled patients with pSS who were admitted to the Department of Rheumatology at Yueyang Hospital of Integrated Traditional Chinese and Western Medicine, Shanghai University of Traditional Chinese Medicine between October 2017 and January 2022**.**

Inclusion criteria for participants were as follows: 1) age over 18 years old; 2) meeting the 2012 American College of Rheumatology (ACR) recommended SS classification criteria (Shiboski et al., 2012); 3) presence of respiratory symptoms or lung abnormalities on HRCT; 4) at least one HRCT examination within 1 year.

The exclusion criteria were as follows: 1) secondary dry syndrome or combined with other rheumatic diseases; 2) combined pulmonary infections, chronic obstructive pulmonary disease, pneumoconiosis and lung diseases such as tumors, tuberculosis, and pulmonary nodules; 3) patients with severe primary diseases of the heart, lungs, liver, kidneys and other organs; 4) patients with incomplete clinical information. Then all patients were divided into pSS-ILD group and pSS-non-ILD group based on whether their HRCT features showed ILD or not. pSS patients whose imaging features supporting ILD in HRCT (e.g., ground-glass opacity, reticulation, consolidation, nodules, traction bronchiectasis, honeycombing) were diagnosed as pSS-ILD (Lohrmann et al., 2004). Altogether 256 pSS patients were screened in this study and divided into the pSS-ILD (102 cases) and pSS-non-ILD groups (154 cases). This study was approved by the Ethics Committee of Shanghai University of Traditional Chinese Medicine (No. 2018-014) and was registered at Chinese Clinical Trial Registry (No. ChiCTR2000037057).

### Data collection

Demographic and clinical data were collected from patients’ medical records, including age, sex, disease duration. Clinical features associated with pSS and pSS-ILD were fully recorded as well, such as xerostomia, xerophthalmia, cough, shortness of breath. Moreover, the blood biochemical data were also collected, including complete blood cell count, erythrocyte sedimentation rate (ESR), C-reactive protein (CRP), blood lipids, rheumatoid factor (RF), immunoglobulin, complement 3 (C3), complement 4 (C4), antinuclear antibody (ANA) spectrum, cytokine.

Blood biochemical data testing methods: Complete blood cell count: The BC-6800 automatic hematology analyzer (mindray, China) was used for testing. The mindray series analyzer’s auto hematology analyzer reagent (mindray, China) was used.

Erythrocyte sedimentation rate (ESR): The TEST 1 Automatic Rapid Blood Sedimentation Analyzer (Alifax, Italy) was used for determination. EDTA anticoagulant was used for blood collection.

C-reactive protein (CRP): The high-sensitivity human C-reactive protein detection kit (mindray, China) was used for the determination of C-reactive protein in human whole blood samples by latex-enhanced immunoscattering turbidimetric assay.

Blood lipids: Enzyme method was used for testing, including total cholesterol (TC), triglycerides (TG), high-density lipoprotein (HDL), and low-density lipoprotein (LDL) testing. Reagent brands: glucose oxidase-cholesterol dehydrogenase method TG kit, glucose oxidase-peroxidase method TC kit, Direct HDL-cholesterol (HDL-c) assays Kit and Direct LDL-c kit (Beckman, United States).

Rheumatoid factor (RF): The N Latex RF Kit (Siemens healthineers, Germany) was used for testing.

Immunoglobulin (Ig), complement 3 (C3), complement 4 (C4): The Scattering turbidimetric method was used for testing. The reagent kit brand was N Antiserum to Immunoglobulin A (IgA), immunoglobulin M (IgM), immunoglobulin G (IgG), complement C3 and complement C4 reagent kit (Siemens healthineers, Germany).

Antinuclear antibody (ANA) spectrum: The EUROLINE ANA profile 3 (Euroimmun Medizinische Labordiagnostika, Germany) was used for testing.

Cytokines: Luminex^®^ xMAP (Luminex Corporation, United States) technology was used for quantitative detection of tumor necrosis factor-α (TNF-α), Interleukin 2 receptor (IL-2R), IL-6 and IL-8. The reagent kit brand was 12 Human Cytokine assay kit (RAISECARE, China).

In addition, all patients underwent HRCT examination. Approach to Radiological Analysis: The Philips iCT 256 spiral computed tomography (CT) scanner was used for image analysis. The tracheal level was selected as the starting position, and the scan was conducted downwards until the bottom of the lungs (excluding the lung apex). The scan parameters were as follows: pitch 1, reconstruction slice thickness of 1.0 mm, interlayer spacing of 1.0 mm, tube current of 40–120 mA, voltage of 120 KV, and reconstruction with a wide window level setting of 2000 HU/-600 HU. All imaging data were reviewed by two experienced radiologists in batches. For high-resolution CT images, a qualitative analysis method and reproducible calculated measurement parameters, including the morphology, extent, and severity of interstitial lung disease (such as linear, nodular, reticular, honeycombing, traction bronchiectasis lesions) were used for classification, counting, and scoring. The maximum diameter and area of all lesions were measured, and the CT density value (CT value) was calculated to evaluate the degree and type of lesion. During the analysis process, independent assessments were performed first, followed by a consensus reached through discussion.

For HRCT abnormalities, different HRCT features of pSS-ILD patients were image-typed according to the American Thoracic Society/European Respiratory Society (2013) International Multidisciplinary Classification of idiopathic interstitial pneumonia (Travis et al., 2013), including Non-specific interstitial pneumonitis (NSIP), Unusual interstitial pneumonia (UIP), Lymphocytic interstitial pneumonitis (LIP), Organizing pneumonia (OP). Additionally, we refer to the literature to stage the severity of fibrosis by chest HRCT in pSS-ILD patients into stages 1, 2, and 3 (Qiu et al., 2008). Stage 1 is defined as early or active lesion with ground glass shadow, increased interlobular stroma and thickened interlobular septa; stage 2 is defined as progressive or chronic prolonged lesion with stage 1 manifestations and interface signs, subpleural arcuate shadow, intrapulmonary lamellar solidity and small nodular shadow; stage 3 is defined as advanced lesion with stage 1, stage 2 and reticular shadow, fibrous streak shadow, honeycomb shadow, multiple large alveoli, distended bronchial dilatation and diffuse fibrosis. Determination of HRCT results were determined by both radiologists and rheumatologist.

### Statistics

All data were statistically analyzed using SPSS 24.0 statistical software. Continuous variables were presented as mean ± standard deviation (SD) for normally distributed data; if the variance had chi-square, two independent samples t-test was used, and if the variance was not chi-square, approximate t-test was used. Median (interquartile spacing) was used for non-normally distributed data and non-parametric test was used for statistical analysis. Categorical variables were presented as the percentages and were compared using chi-square test or Fisher’s exact test. Logistic regression analysis was used to identify the factors associated with the development of ILD in patients with pSS. All reported *p*-values were two sided and *p* < 0.05 was considered to be statistically significant.

## Results

### Demographic and clinical features in patients with pSS-interstitial lung disease (ILD)

This study included a total of 256 patients with pSS. Of these, there were 102 patients in the pSS- ILD group, with a mean age of 63.36 ± 9.22 years, of whom 66 (64.71%) were over 60 years old. The pSS-non-ILD group consisted of 154 patients, with a mean age of 57.16 ± 12.72 years, of whom 73 (47.40%) were over 60 years old. Generally, there were significant differences between the pSS-ILD and total pSS-non-ILD groups related to age (*p* < 0.01) and disease duration (*p* < 0.001) characteristics studied (Table 1).

**TABLE 1:** Demographics and clinical characteristics of the primary Sjögren’s syndrome (pSS)- interstitial lung disease (ILD) and pSS-non-ILD.

	pSS-ILD (*n* = 102)	pSS-non-ILD (*n* = 154)	*p*-value
Demographics			
Sex (male, %)	4 (3.92%)	12 (7.79%)	0.210
Age (years)	63.36 ± 9.22	57.16 ± 12.72	0.000*
Disease duration (months)	90 (24, 156)	48 (24, 120)	0.006*
Clinical symptoms			
Xerostomia (*n*, %)	90 (88.24%)	137 (88.96%)	0.858
Xerophthalmia (*n*, %)	87 (85.29%)	115 (74.68%)	0.041*
Arthralgia (*n*, %)	44 (43.14%)	62 (40.26%)	0.647
Fever (*n*, %)	18 (17.65%)	23 (14.94%)	0.562
Fatigue (*n*, %)	18 (17.65%)	23 (14.94%)	0.359
Raynaud’s phenomenon (*n*, %)	18 (17.65%)	23 (14.94%)	0.606
Cough (*n*, %)	7 (6.86%)	9 (5.84%)	0.742
Cough and sputum (*n*, %)	7 (6.86%)	9 (5.84%)	0.000*
Chest tightness (*n*, %)	7 (6.86%)	9 (5.84%)	0.050
Shortness of breath (*n*, %)	23 (22.55%)	4 (2.60%)	0.000*
Skin rash (*n*, %)	23 (22.55%)	4 (2.60%)	0.553
Myalgia (*n*, %)	4 (3.92%)	8 (5.19%)	0.768^a^
Laboratory metrics			
ESR (mm/h)	24.5 (13.75, 49.25)	20 (11, 75)	0.044*
CRP elevation (n, %)	23 (22.55%)	20 (12.99%)	0.045*
WBC (×10^9^/L)	5.40 (4.10, 7.03)	5.10 (3.68, 6.30)	0.027*
PLT (×10^9^/L)	183.00 (153.00, 226.00)	182.50 (137.00, 233.25)	0.888
Hb (g/L)	123.08 ± 13.42	120.14 ± 13.69	0.091
NLR	2.49 (1.65, 4.13)	2.13 (1.50, 3.17)	0.044*
TG (mmol/L)	1.28 (0.96, 1.84)	1.12 (0.84, 1.51)	0.004*
TC (mmol/L)	4.80 (3.90, 5.49)	4.50 (3.70, 5.10)	0.046*
HDL (mmol/L)	1.22 (0.99, 1.48)	1.22 (1.02, 1.44)	0.828
LDL (mmol/L)	2.93 (2.36, 3.40)	2.75 (2.32, 3.26)	0.099
RF^+^ (*n*, %)	32 (31.37%)	36 (23.38%)	0.193
ANA^+^ (*n*, %)	87 (85.29%)	133 (86.36%)	0.810
Anti-Ro52^+^ (*n*, %)	65 (63.73%)	77 (50.00%)	0.040*
Anti-Ro60^+^ (*n*, %)	65 (63.73%)	102 (66.23%)	0.690
Anti-SSB^+^ (*n*, %)	39 (38.24%)	42 (27.27%)	0.075
Anti-centromere^+^ (*n*, %)	9 (8.82%)	32 (20.78%)	0.014*
C3 (g/L)	0.89 (0.78, 1.00)	0.89 (0.77, 1.02)	0.896
C4 (g/L)	0.195 (0.15, 0.24)	0.20 (0.15, 0.24)	0.528
IgG (g/L)	13.95 (11.28, 18.20)	14.25 (11.55, 17.30)	0.948
IgM (g/L)	0.80 (0.60, 1.16)	0.88 (0.63, 1.22)	0.386
IgA (g/L)	2.65 (1.92, 3.75)	2.46 (1.72, 3.42)	0.163
IL-2R (u/mL)	507.00 (375.50, 645.30)	452.50 (355.50, 591.00)	0.175
IL-6 (pg/mL)	3.32 (2.00, 6.23)	2.39 (2.00, 4.16)	0.030*
IL-8 (pg/mL)	28.80 (17.80, 57.30)	28.60 (16.80, 59.80)	0.717
TNF-α (pg/mL)	10.00 (7.70, 12.70)	9.50 (7.40, 11.70)	0.271

ESR, erythrocyte sedimentation rate; CRP, C-reactive protein; WBC, white blood cell count; PLT, platelets; NLR, neutrophil/lymphocyte ratio; Hb, hemoglobin; TG, triglycerides; TC, total cholesterol; HDL, high-density lipoprotein; LDL, low-density lipoprotein; RF, rheumatoid factor; ANA, antinuclear antibody; C3, complement 3; C4, complement 4; IgG, immunoglobulin G; IgM, immunoglobulin M; IgA, immunoglobulin A; IL-2R, interleukin-2 receptor; IL-6, interleukin-6; IL-8, interleukin-8; TNF-α, tumor necrosis factor-α. *, The difference between the groups was statistically significant. ^a^, denotes calculation using Fisher’s exact probability method.

The meaning of the bold values indicates statistically significant differences.

Next, clinical symptoms of the two groups were analyzed. Among 256 patients with pSS, the most common clinical manifestations were xerostomia, xerophthalmia, and arthralgias. Fever, fatigue, Raynaud’s phenomenon, cough and sputum, chest tightness and shortness of breath, skin rash, muscle aches and pains have been observed in a few patients. Patients with pSS-ILD have a significantly higher proportion of clinical manifestations of xerophthalmia (85.2%), cough (25.4%), and shortness of breath (22.5%) compared with patients without ILD (*p* < 0.05) (Table 1).

### Laboratory metrics

As shown in Table 1, ESR, CRP, white blood cell count (WBC), neutrophil/lymphocyte ratio (NLR), TC, and TG were significantly higher in the pSS-ILD group than in the pSS-non-ILD group (*p* < 0.05). As for the immunologic index, the positivity for different autoantibodies in pSS-ILD groups was as follows: 87 cases (85.29%) for ANA, 65 cases (63.73%) for Ro52, 65 cases (63.73%) for Ro60, 39 cases (38.24%) for SSB, and 9 cases (8.82%) for anti-centromere antibodies. By chi-square test, patients in the pSS-ILD group had a higher rate of anti-Ro52^+^ and a lower rate of anti-centromere^+^ than those in the pSS-non-ILD group, and the differences between the two groups were statistically significant (*p* < 0.05) (Table 1).

In addition, among all pSS patients, cytokines were measured and analyzed in 222 patients showed that IL-6 was significantly higher in the pSS-ILD group than in the pSS-non-ILD group (*p* < 0.05), while no statistically significant differences between the remaining cytokines in the two groups (*p* > 0.05) (Table 1).

### HRCT features

HRCT provides superior evaluation of the lung compared with conventional radiographs. If HRCT findings are characteristic, lung biopsy is not necessary to confirm ILD in humans. In our study, all 256 patients underwent HRCT and 102 of them showed interstitial lung lesions on HRCT. For HRCT abnormalities, the percentages of different patterns were showed in Table 2. Of note, ground glass-like shadow described as a hazy increased opacity of the lungs was the most common, followed by patchy solid shadow. The percentages of different stages were as follows: 38 patients (34.3%) were in stage 1 (early stage), 35 patients (34.3%) were in stage 2 (chronic prolongation), and 29 patients (28.4%) were in stage 3 (late stage). And then HRCT imaging classification of pSS-ILD patients were showed as follows: 74 cases (72.5%) with NSIP, 10 cases (9.8%) with UIP, 6 cases (5.9%) with LIP, and 2 cases (2.0%) with OP. However, due to atypical HRCT imaging manifestations that could not be clearly defined to which imaging type they belonged, or the presence of two or more types of imaging manifestations at the same time, they were categorized as other in our study results (10 cases, 9.8%) (Table 2).

**TABLE 2:** Clinical characteristics of patients with primary Sjögren’s syndrome (pSS)-interstitial lung disease (ILD).

	Number of patients (%)
Subjects	102 (39.84)
Gender, male	4 (3.92)
respiratory symptoms	51 (50.00)
HRCT features	
Ground-glass opacity	80 (78.43)
Reticulation	18 (17.65)
consolidation	18 (17.65)
Honeycombing	3 (2.94)
NSIP	74 (72.55)
UIP	10 (9.80)
LIP	6 (5.88)
OP	2 (1.96)
Stage 1	38 (37.3)
Stage 2	35 (34.3)
Stage 3	29 (28.4)

NSIP, non-specific interstitial pneumonia; UIP, usual interstitial pneumonia; LIP, lymphocytic interstitial pneumonia; OP, organizing pneumonia.

**FIGURE 1:**
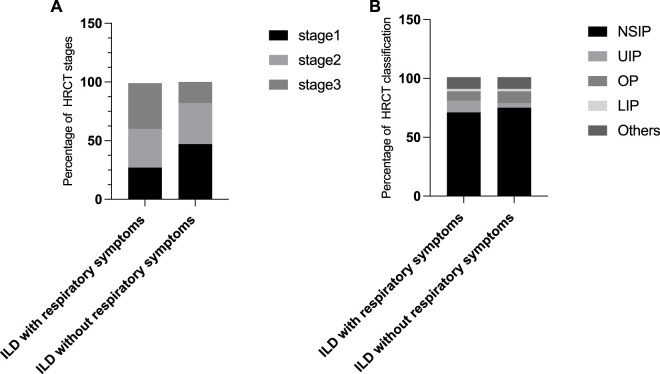
Characteristics of HRCT in pSS-ILD patients with or without respiratory symptoms. **(A,B)** Percentage of different HRCT staging **(A)** and classification **(B)** in ILD patients with and without respiratory symptoms.

Additionally, we investigated the features for ILD detected by HRCT in pSS patients with or without respiratory symptoms. It is noteworthy that 50 percentage of patients without obvious pulmonary symptoms exhibited abnormalities, consistent with ILD symptoms, in HRCT-scan. We further observed the characteristics of HRCT in these patients without respiratory symptoms (imaging performance, staging and classification). Obviously, HRCT features in pSS-ILD patients without obvious pulmonary symptoms are predominantly early compared to patients with obvious pulmonary symptoms (Figure 1). This suggests that regular HRCT should also be performed in patients with pSS without respiratory symptoms.

### Risk factors for the development of ILD and predictive model

Furthermore, we also performed logistic regression analysis to investigate the independent risk factors of interstitial lung lesions in patients with pSS. In the univariate logistic regression analysis comparing the pSS-ILD cohort with the control cohort, several variables showed statistically significant odds ratios. The pSS-ILD cohort had an odds ratio (OR) of 2.034 (95% CI 1.216–3.414, *p* = 0.007) for age>60 years, 1.006 (95% CI 1.003–1.010, *p* < 0.001) for disease duration, 0.103 (95% CI 0.024–0.446, *p* = 0.002) for shortness of breath. Among the laboratory metrics, the following ORs were observed: ESR (OR = 1.010, 95% CI 1.000–1.020, *p* = 0.046); CRP (OR = 1.951, 95% CI 1.008–3.776, *p* = 0.047); WBC (OR = 1.150, 95% CI 1.032–1.281, *p* = 0.011); NLR (OR = 1.179, 95% CI 1.043–1.333, *p* = 0.008); TG (OR = 1.793, 95% CI 1.191–2.700, *p* = 0.005); TC (OR = 1.329, 95% CI 1.038–1.702, *p* = 0.024); Anti-Ro52^+^ (OR = 1.949, 95% CI 1.167–3.255, *p* = 0.011); Anti-centromere^+^ (OR = 0.369, 95% CI 0.168–0.811, *p* = 0.013).

Based on the results and clinical relevance, 11 variables were a step forward included in the multivariate logistic regression analysis. The finally results showed that older than 60 years (OR = 2.381, 95% IC 1.302–4.355, *p* < 0.05), longer duration of disease (OR = 1.007, 95% IC 1.003–1.012, *p* < 0.001), higher TG (OR = 1.652, 95% CI 1.014–2.691, *p* < 0.05), higher TC (OR = 1.424, 95% CI 1.059–1.915, *p* < 0.05) and anti-Ro52^+^ (OR = 1.949, 95% CI 1.167–3.255, *p* = 0.011) were risk factors for ILD in pSS patients. Interestingly, we also found a protective factor for ILD in pSS patients was anti-centromere^+^ (OR = 0.402, 95% CI 0.17–0.935, *p* < 0.05) (Table 3).

**TABLE 3:** Possible relevant risk factors of developing interstitial lung disease among primary Sjögren’s syndrome (pSS) patients.

Characteristics	Univariate			Multivariate		
	Odds ratio	95% CI	*p*-value	Odds ratio	95% CI	*p*-value
Gender, male	0.483	0.151–1.541	0.219			
Age>60 (years)	2.034	1.216-3.414	0.007**	2.035	1.140–3.634	0.016*
Disease duration (months)	1.006	1.003–1.010	0.000***	1.006	1.002–1.010	0.002**
Xerostomia	1.304	0.580–2.934	0.520			
Xerophthalmia	0.866	0.471–1.592	0.642			
Shortness of breath	0.103	0.024–0.446	0.002**			
Chest tightness	0.657	0.341–1.256	0.209			
ESR (mm/h)	1.010	1.000–1.020	0.046*	1.003	0.988–1.107	0.711
CRP elevation	1.951	1.008–3.776	0.047*	1.902	0.662–5.461	0.232
WBC (×10^9^/L)	1.150	1.032–1.281	0.011**	1.045	0.908–1.203	0.538
NLR	1.179	1.043–1.333	0.008**	1.038	1.002–1.010	0.660
TG (mmol/L)	1.793	1.191–2.700	0.005**	1.664	1.020–2.714	0.041*
TC (mmol/L)	1.329	1.038–1.702	0.024*	1.349	1.004–1.812	0.047*
HDL (mmol/L)	0.905	0.426–1.923	0.795			
LDL (mmol/L)	1.413	0.998–1.999	0.051			
Anti-Ro52^+^	1.949	1.167–3.255	0.011*	1.812	1.002–3.287	0.049*
Anti-Ro60^+^	0.896	0.530–1.512	0.680			
Anti-SSB^+^	1.651	0.968–2.816	0.066			
Anti-adnexin^+^	0.369	0.168–0.811	0.013*	0.405	0.174–0.945	0.037*
RF^+^	1.498	0.855–2.625	0.157			
ANA^+^	1.076	0.613–1.890	0.799			

NLR, neutrophil–lymphocyte ratio; LDL, low-density lipoprotein; TC, total cholesterol; TG, triglyceride; RF, rheumatoid factor; ANA, antinuclear antibodies. *p < 0.05; **p < 0.01; ***p < 0.001.

The meaning of the bold values indicates statistically significant differences.

## Discussion

The pathogenesis of pSS-ILD is not fully elucidated. An early study revealed that the combined involvement of genetic, environmental and infection susceptibility factors result in recruitment of inflammatory cells into the interstitial and alveolar spaces of the lung, releasing inflammatory factors and causing alveolar epithelial damage. Consequently, fibroblasts and myofibroblasts in the interstitial spaces of the lung become activated and produce extracellular matrix proteins, leading to lung fibrosis (Wells and Denton, 2014; Panagopoulos et al., 2021). In our study, patients in the pSS-ILD group had significantly higher WBC counts than patients in the pSS-non-ILD group. WBC, as an inflammatory cell, can activate inflammatory responses in certain pathological conditions, so is it possible that patients in the pSS-ILD group had higher inflammatory indicators? We then further analyzed the inflammatory indexes in both groups and found that CRP, ESR, NLR, and IL-6 were significantly higher in the pSS-ILD group than that in the patients without ILD. NLR, as a new inflammatory index that can reflect the systemic inflammatory status, is commonly used in the diagnosis and assessment of prognosis of infectious diseases. It includes lymphocyte and neutrophil counts, which has the advantages of being simple, easy to operate, inexpensive and reproducible. And it is more clinically meaningful than single lymphocyte and neutrophil counts, and can be widely used in clinical practice to assess the inflammatory status of diseases in combination with CRP, ESR and other indexes. IL-6 is a multifunctional cytokine with multiple immunomodulatory effects, and its elevation is positively correlated with inflammation. Previous study (Boumba et al., 1995; Mavragani, 2017) reported that IL-6, IL-8, IL-10, and TNF-α may be involved in the occurrence and development of ILD in patients with pSS, suggesting that the occurrence of pSS-ILD is closely related to the inflammatory response and needs clinical attention.

In addition, pSS also can attack blood lipids and promote the development of atherosclerosis (Vaudo et al., 2005). Previous study reported that pSS patients had a strong trend to present dyslipidemia when compared to healthy individuals (Cruz et al., 2010). Consistently, our study showed that TG and TC were significantly higher in the pSS-ILD group than in the pSS-non-ILD group. As for the reasons, the elevation of TG and TC may be related to the older age of patients. On the other hand, it also suggested that pSS-ILD patients were more likely to have abnormal TG and TC than patients without ILD. In clinical treatment, we need to pay more attention to and monitor the lipid profile of patients in order to reduce the risk of cardiovascular and cerebrovascular adverse events in patients. Certainly, glucocorticoid can also cause dyslipidemia in patients, which could be a potential confounding factor.

Moreover, immunological factors also play an important role in the pathogenesis of pSS-ILD, and multiple immune cells are jointly involved in the pathogenesis (Ytterberg et al., 2015). We further analyzed the immunological indicators between the two groups, and the results showed that the anti-RO52^+^ rate in pSS-ILD group was significantly higher than that in non-ILD group, while the positive rates of immunoglobulin and complement were not significantly different, indicating that anti-Ro52^+^ pSS patients were more prone to ILD. Davidson et al. (2000) have reported that lung involvement occurs primarily in pSS patients who are positive for anti-RO antibodies and can occur early in the disease. This suggests that early screening should be conducted for such patients in clinical practice.

pSS-ILD is asymptomatic and easy to be missed, and some patients have no obvious clinical symptoms in the early stage. HRCT can detect abnormal changes in the lungs of pSS patients at an early stage with a specificity of 90%, which is the same as the pathological findings of lung biopsy. In this study, 256 patients underwent HRCT of the lung, and 102 of them showed interstitial lung lesions, which mainly consisted of ground glass-like shadow, patchy solid shadow, fibrous streak shadow, lattice shadow and honeycomb shadow, among which ground glass-like shadow was the most common, followed by patchy solid shadow. HRCT staging of patients based on imaging presentation revealed that NSIP was the most common type of ILD in patients with pSS. Abrasive glassy shadow is an early manifestation of interstitial lung changes, mostly reversible, while lattice-like and honeycomb-like changes are indicative of intermediate and advanced stages of irreversible fibrosis. In addition, it is noteworthy that 50% patients without obvious pulmonary symptoms exhibited abnormalities, consistent with ILD features in HRCT-scan, although these were relatively early. Therefore, HRCT can help to diagnose the disease at an early stage so that we can better grasp the timing of treatment. In addition, respiratory symptoms appeared later than pulmonary imaging findings in some patients, which requires clinicians to judge whether there is pulmonary involvement in pSS patients, not only based on respiratory symptoms, but also regular imaging examinations to achieve the purpose of early diagnosis.

Pulmonary involvement increases the 10-year mortality of pSS patients by 4 times (Palm et al., 2013). Early diagnosis and treatment can prevent pulmonary lesions from progressing from reversible inflammatory stage to irreversible pulmonary fibrosis stage, and significantly improve the prognosis of patients. After single factor screening, eleven clinical variables were found to be significantly different between two groups. These factors were age, course of disease, CRP, ESR, WBC, NLR, TG, TC, anti-Ro52^+^, anti-centromere^+^, and IL-6. The multivariate analysis showed that age ≥60 years, chronic disease, high TG and high TC were the high risk factors for ILD in pSS patients, which was consistent with previous studies (Gao et al., 2018). Analysis of the reason may be induced in older adults with the body immune function change matter more, thus easier to cause the change of immune, eventually lead to the occurrence of ILD. Surprisingly, our study also found that anti-centromere^+^ is a protective factor for ILD, which is similar to what has been reported in previous studies, the antibodies against the centromere has protective effect to the organs in the diffuse systemic sclerosis (dsSSc) (Caetano et al., 2018). However, in our literature review, we found no reports of anti-Ro52^+^ and ILD in pSS patients. The correlation between the level of autoimmune antibodies in pSS patients and the incidence of ILD is worthy of further investigation.

In addition, there were some limitations in our study. First of all, this study was a retrospective, single-center, and small-scale study, which may affect the universality and practical clinical significance of the results. Second, there was a correlation between some of the indicators, such as high age and elevated lipid levels, which may have led to potential confusion. Third, some results with statistical differences lacked appropriate clinical interpretation, which affected the authenticity of the results. In the next step, we will conduct a multi-center study with a large sample size and collect more comprehensive patient clinical data to reduce controllable interference factors and provide advantages for early diagnosis, treatment and prognosis of pSS-ILD.

## Conclusion

The clinical features of pSS-ILD patients were xerophthalmia, cough and shortness of breath, and HRCT can help to diagnose the disease at an early stage. Age over 60 years, chronic course of disease, and elevated lipid levels are risk factors for ILD in pSS patients, and the relationship between autoimmune antibody levels and the occurrence of ILD needs to be further confirmed in follow-up studies with large sample sizes.

## Data Availability

The raw data supporting the conclusion of this article will be made available by the authors, without undue reservation.
